# Ovarian Endometriosis and Adenomyosis—Relevance, Pathophysiology of Ectopic Endometrium and Impact on Dysfunction of Eutopic Endometrium: A Narrative Review

**DOI:** 10.3390/biomedicines14061343

**Published:** 2026-06-13

**Authors:** Liudmila M. Mikhaleva, Mekan R. Orazov, Evgeny D. Dolgov, Sergey A. Mikhalev, Zarina V. Gioeva, Alexander V. Ilyichev, Natalia B. Tikhonova, Lolita S. Bulatova

**Affiliations:** 1 Federal State Budgetary Scientific Institution “Russian Scientific Center of Surgery Named After Academician B.V. Petrovsky”, Scientific Research Institute of Human Morphology Named After Academician A.P. Avtsyn, 117418 Moscow, Russia; mikhalevalm@yandex.ru (L.M.M.); 1586dolgde@gmail.com (E.D.D.); alex730110@gmail.com (A.V.I.); nb-ti@hotmail.com (N.B.T.); lolita.bulatova.87@mail.ru (L.S.B.); 2 Institute of Medicine of the Federal State Autonomous Educational Institution of Higher Education «Peoples’ Friendship University of Russia», Department of Obstetrics and Gynecology, 117198 Moscow, Russia; omekan@mail.ru; 3 Federal State Autonomous Educational Institution of Higher Education “N.I. Pirogov Russian National Research Medical University” of the Ministry of Health of the Russian Federation, Research Laboratory of Women’s, Maternal and Child Health, 117997 Moscow, Russia; mikhalev@me.com

**Keywords:** endometriosis, endometrioid ovarian cysts, adenomyosis, pathogenetic pathways, endometrium, endometrial dysfunction, implantation failure, endometrial implantation failure

## Abstract

A trend toward comorbid conditions is seen in around 50% of gynecological patients, with a significant contribution made by endometriosis as a common and incurable gynecological condition. Over the last decades, the global burdens of different forms of endometriosis have shown a progressive increase, while their diagnosis and management present persistent and significant challenges. Currently, endometriosis is divided into two primary types: genital (adenomyosis and external genital endometriosis, including ovarian endometriosis) and extragenital endometriosis. Regardless of the location of endometriosis, lesions or ectopic endometrium follow a consistent pathological process characterized by active proliferation, local inflammation, neoangiogenesis, and extracellular matrix remodeling. These pathogenetic patterns are associated not only with process progression, but also with the impact on the eutopic endometrium. External genital or extragenital endometriosis and adenomyosis (an internal genital endometriosis) are currently considered as a major cause of infertility and implantation failures due to the negative impact on the eutopic endometrium. However, it has been proven that the pathogenetic pathways for the development of eutopic endometrium dysfunction in these endometriosis phenotypes (despite the common pathophysiology of the ectopic endometrium) differ significantly. This narrative review is focused on highlighting the relevance and pathogenetic patterns of the two most frequently diagnosed forms of endometriosis—adenomyosis and ovarian endometrioid cysts—as key areas of research interest relating to their relevance, specific pathophysiology and impacts on the eutopic endometrium.

## 1. Introduction

Endometriosis is recognized as one of the common gynecological conditions. It is often associated with pelvic pain and involves multiple etiological factors contributing to implantation failures caused by the pro-inflammatory microenvironment that significantly disrupts molecular biological homeostasis. Implantation failures in women with endometriosis are also linked to endometrial dysfunction, which may be caused by the distant impact of pathogenetic molecular messengers and various genetic and epigenetic aberrations.

A common etiopathology for adenomyosis and endometriosis as ectopic endometrial lesions has been suggested because both conditions entail the presence of endometrial tissue at locations other than the lining of the uterus. However, despite the similarities in the pathophysiology of the ectopic endometrium (endometrioid cysts and adenomyomas), these two conditions have very different effects on the dysfunction of the eutopic endometrium. Regarding connection, the goal of this article is to review the relevance and pathogenesis of ovarian endometriosis and adenomyosis as the most prevalent phenotypes of endometriosis, acting as top research priorities, and having differences in specific endometrial dysfunction factors. Also, an in-depth analysis is performed to elucidate complex pathogenetic interactions between the endometrium, endometriotic ovarian cysts (EOCs) and adenomyosis (one of the most frequent and significant endometriosis phenotypes), as these conditions are associated with pathological processes in the eutopic endometrium.

## 2. Methods

This study employs a narrative review approach. We selected literature with strong thematic relevance and significant academic influence from English databases to support an in-depth discussion of the core issues. This review article presents a comprehensive analysis of the recent literature, highlighting the relevance of the topic, pathogenetic aspects of ovarian endometriosis and adenomyosis, and their association with endometrial dysfunction. We conducted a search using the databases PubMed, Web of Science and Google Scholar, covering articles published up to 25 December 2025. Search terms included ‘endometriosis,’ ‘endometrioid ovarian cysts,’ ‘ovarian endometriosis,’ ‘adenomyosis, ‘pathogenetic pathways,’ ‘endometrial dysfunction,’ ‘genetic changes,’ ‘epigenetic changes,’ and ‘endometrial implantation failure’.

Based on a consensus among all authors, the inclusion and exclusion criteria were established. Eligible studies were required to meet the following criteria: (a) published in the Russian or English languages; (b) original full-text articles; and (c) focused on the pathogenesis of ovarian endometriosis/adenomyosis and their association with endometrial dysfunction. Additionally, citations from the selected studies were analyzed with the aim of including more relevant publications. Studies were excluded if they were: (a) not in English or Russian; (b) non-original research; (c) withdrawn articles; (d) not available in full text; or (e) not directly related to the study focus (ovarian endometriosis, adenomyosis, or endometrial dysfunction). Ultimately, 67 scientific publications were included in our article.

## 3. Results

### 3.1. Ovarian Endometriosis

#### 3.1.1. Definition and Epidemiology

Endometriosis is a chronic, continuously progressing, inflammatory, estrogen-dependent disease marked by the presence of tissue that is similar, by its morphological and functional parameters, to the eutopic endometrium. The disease is associated with variable clinical presentation and debilitating painful symptoms, affects social functioning and often leads to a decrease in women’s quality of life [1,2,3]. Nowadays, endometriosis is still considered an incurable gynecological disease, having one of the longest diagnostic times, often taking an average of 7 to 10 years from the onset of symptoms to a confirmed diagnosis. Over the diagnostic delay, the pathogenetic mechanisms of endometriosis significantly enhance, and later, following the surgical excision of heterotopic tissues, patients can still experience algological symptoms (due to long-term central sensitization, disease recurrence, residual heterotopia fragments, and ill-advised post-surgical care) [4,5].

Recent epidemiological data indicate that endometriosis is estimated to affect 10% of reproductive-age women, which extrapolates to approximately 190 million women worldwide [6,7]. Moreover, the analysis of endometriosis trends over the last several decades has revealed a consistent increase in prevalence [8,9,10]. A recent Japanese study on the epidemiology of endometriosis which included 4.9 million women demonstrated that at 15–50 years of age, the hypothetical cumulative incidences were 37.34% for overall endometriosis. With that, the authors also pointed out that the prevalence of endometriosis increased consistently [11]. It is important that for the most common clinical type of endometriosis, ovarian endometriosis, the ovarian endometriotic cysts (endometriomas) act as a substrate. It is estimated that 17 to 44% of women with endometriosis experience ovarian endometrioma, and 28% of these women will have bilateral ovarian endometriomas [12,13]. Moreover, recent studies have highlighted that approximately 80% of patients with endometriosis suffer from ovarian endometriosis [14].

**Figure 1:**
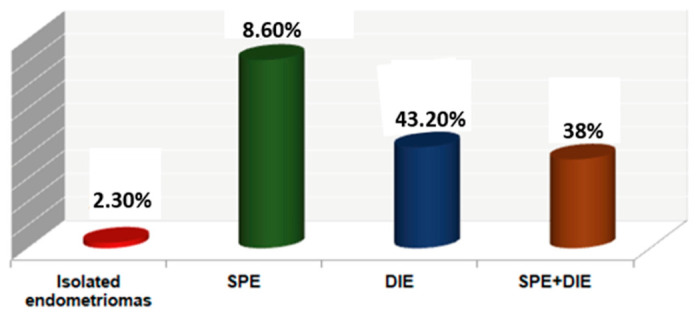
Frequency of diagnosing coexisting types of ovarian endometriosis [15].

It should be noted that ovarian endometriomas are rarely an isolated finding. In a study including over 1000 patients, endometriomas were alone in only 2.3% of the cases. In the other patients, endometriomas occurred concurrently with other endometriosis types: in 80.6%—with superficial peritoneal endometriosis (SPE), in 43.2%—deep infiltrating endometriosis (DIE), and in 38% of the patients—with both SPE and DIE (Figure 1) [15].

#### 3.1.2. Pathogenesis Pathways

According to the definition (see above), endometriosis is widely recognized as a disease directly dependent on the local and systemic levels of estrogen and a pro-inflammatory environment in the tissues. However, its pathogenesis involves a hierarchy of factors, creating a complex interplay of the “top-level” pathogenetic drivers that can lead to both estrogen dependence and inflammation and cause the subsequent impairments. Since ovarian endometriosis frequently coexists with SPE and DIE, the co-occurrence and distinct nature of these two phenotypes may induce specific pathogenetic disorders. The current pathogenetic concept of endometriosis involves a hierarchy of factors that build upon one another.

**(1) Epigenetic factors**. The specific changes in histone conformation, impaired DNA methylation patterns (*GATA2, GATA6, SF-1*), and a significant misbalance of regulating non-coding microRNAs (miRNAs) have been established in patients with endometriosis [16]. Recent research of Nahdi S. et al. (2025) has shown that the levels of miR-146a-5, which plays a role in immune modulation and inflammatory signaling, were significantly upregulated and associated with disease progression in patients with diagnosed endometriomas [17]. It is also worth referring to the study conducted by Sun L. et al. (2024), which identified the key exosomal microRNA miR-21-5p as activating angiogenesis in endometriotic lesions [18]. Its effect is achieved by activating endothelial cell migration and proliferation and, therefore, may contribute to the progression of endometriotic lesions [18].

**(2) Genetic factors.** Current research estimates that approximately 50% of the risk for developing endometriosis is attributable to genetic factors [19]. In patients with endometriosis (including the ovarian type), pathogenesis is associated with the impaired expression of various regulatory genes:

- A decrease in the expression levels of *RASA1* and *NF1* genes: The *RASA1* gene plays a role in controlling the local angiogenesis process (an important factor in the progression of heterotopias), and the *NF1* gene promotes the pro-apoptotic potential of cells [20,21,22].
- An increase in the expression level of claudin (*CLDN*) family genes (3, 5, 7, 11) and Homeobox family genes C (*HOXC6*, *HOXC8*) and B (*HOXB6*, *HOXB7*): Claudins are crucial components of tight junctions, which are key to intercellular adhesion, and HOX genes are essential for endometrial development, differentiation, and receptivity [23,24,25].
- Altered expression of apoptosis-regulating genes: Recent research of Soykan Y. et al. (2025) has demonstrated that in the follicular fluid of patients with identified endometriomas, pro-apoptotic genes (*CASP3*, *CASP8*, *CASP9*, *BAX*, *BAK*, *PERFORIN u GRANZYME*) exhibited significantly higher expression levels (*p* < 0.05), while anti-apoptotic genes like *BCL-2* and *BCL2L1* were significantly lower (*p* < 0.05) compared to controls [26]. This study demonstrates that apoptosis markers play a more dominant role in diminished ovarian reserve compared to proliferation inducers in infertile women with ovarian endometriosis. However, these findings are inconsistent with other studies, since endometriosis (of any location) is frequently characterized by a predominance of proliferative components over cell apoptosis. Thus, the results of this study could be specific to the cohort of infertile women, supporting the assertion that molecular changes differ between fertile and infertile endometriosis patients. In this regard, research by Sapmaz, T. et al. (2022), involving animal models of ovarian endometriosis, revealed decreased expression of pro-apoptotic *BAX* expression and increased expression of anti-apoptotic mediator *Bcl-2* compared to native tissue samples [27]. In summary, the imbalance of pro- and anti-apoptotic factors in ovarian endometriosis remains a matter of considerable controversy. As mentioned above, there is a potential linkage between the predominance of apoptotic markers and endometriosis-associated infertility. At the same time, the molecular alterations associated with proliferative factors are key elements of the classic pathogenetic pattern of endometriosis in patients whose fertility is not impaired. Further research is needed to better understand specific mechanisms.

**(3) Hormonal and receptor factors**. The increased local production of estrogens within the endometriotic lesions due to the aberrant activity of the P-450 aromatase enzyme, and the suppression of the 17beta-hydroxysteroid dehydrogenase type 2 (HSD17B2) enzyme, lowering estrogenic effects, have been found in patients with endometriosis. An overexpression of the estrogen receptor beta (ERβ) subtype was established in the endometrial lesions. Also, progesterone resistance in both endometriotic lesions and eutopic endometrium was revealed in women with endometriosis [28,29].

**(4) Disruptions of local tissue homeostasis:**

- *Invasive potential of heterotopias:* The research shows that women with ovarian endometriosis may express high levels of transforming growth factor-beta1 (TGF-β1), which can enhance the invasive potential of cells in endometrioid heterotopias [30].
- *Pro-inflammatory changes:* Endometriosis is associated with significantly increased levels of pro-inflammatory cytokines, such as TNF-α, IL-6, IL-1β, and IL-8. This inflammatory environment, via cyclooxygenase-2 (COX-2) activity, stimulates the expression and activity of the P-450 aromatase enzyme within endometrial tissue, thus closing the vicious cycle of pathogenesis [31]. The study conducted by Nahdi S. et al. (2025) suggests that patients with diagnosed ovarian endometriosis have increased levels of mRNA IL-6, which contributes to local inflammation in the ovarian tissue and impairs the physiological status and quality of oocytes, which then disrupts embryo implantation [17]. The authors revealed that the mRNA IL-6 level correlated with the expression of target genes. The research also showed that the increased level of this pro-inflammatory marker suggests the presence of post-transcriptional regulatory mechanisms that probably act through miRNAs [17].
- *Local autophagy alterations:* Patients with ovarian endometriosis had impaired expression of autophagy gene LC3 and disrupted protein ubiquitination processes that were tightly linked to ovarian health, affecting ovarian reserve, accelerating follicle loss and oxidative damage [17].
- *Enhanced angiogenesis:* Studies have consistently shown that patients with endometriosis exhibit increased expression levels of vascular endothelial growth factor (VEGF). In turn, VEGF contributes significantly to the vascularization of endometrial heterotopias and proliferative cell potential, thus supporting the progression of ectopic endometrial lesions [32]. It has now been proven that angiogenesis in endometriosis is realized through three different mechanisms: the VEGF-A/TGF-β1 axis, the ANG2/ANG1 axis, and the HIF-1α-regulated axis. In a study on ovarian endometriosis cell lines, a significant increase in the expression of factors of each of these pathways was revealed—in particular, VEGF-A mRNA (*p* < 0.0001), the VEGF-A-to-TGF-β1 ratio in 12Z (*p* < 0.0001), ANG2 gene expression (*p* < 0.0001), and the expression of hypoxia-inducible factor-1 (HIF-1α) [33].
- *Hypoxic changes:* It has now been proven that ectopic endometrium, due to its high proliferative activity, is accompanied by the development of local hypoxic changes that induce the release of HIF-1α. This biological messenger is capable of initiating changes in the transcription of genetic loci responsible for angiogenesis, proliferation, survival, and migration of heterotopic cells [34,35]. This is confirmed by the results of a study (2025) that included 87 patients with previous cystectomy for ovarian endometriosis, according to which a significant increase in the expression of HIF-1α (*p* < 0.05) was noted in the cyst capsule and in the foci of ectopic endometrium themselves [36]. Moreover, in patients with ovarian endometriosis, an increase in the systemic level of free oxygen radicals (FORT) and their imbalance with free oxidant radical defense (FORD) (*p* = 0.027) is observed, which reflects the presence of pronounced oxidative stress in the ectopic endometrium, potentiating the progression of heterotopias [37].

In conclusion, the pathogenesis of endometriosis (including the ovarian type) is a complex and multifactorial process, characterized by the hierarchy of impairment levels. It involves an interplay of genetic, molecular, immunological and other factors that act both locally and have distant systemic effects, particularly on the endometrium. This issue is described in more detail below.

### 3.2. Adenomyosis

#### 3.2.1. Definition and Epidemiology

Adenomyosis (historically referred to as “endometriosis interna”) and endometriosis share very similar definitions and pathogenesis pathways, but the key difference is location: adenomyosis is characterized by the presence of ectopic endometrial tissue (both glands and stroma) within the myometrium, forming “invaginations.” Thus, adenomyosis is characterized by the presence of endometrial tissue within the muscular wall of the uterus itself, while in endometriosis, the endometrial-like tissue grows outside the uterus [38]. According to the overall epidemiological data, the prevalence of adenomyosis ranges widely from approximately 9% to 62% in women of reproductive age. According to the results of transvaginal ultrasonography, the overall prevalence in relevant populations generally falls within the range of 12% to 34% [39]. This discrepancy in epidemiological data is primarily attributed to the difficulties in diagnosing this disease and the fact that many cases are subclinical.

#### 3.2.2. Pathogenesis Pathways

The pathogenetic mechanisms and factors involved in the development of endometriosis have been discussed in the above sections of this review. It should be noted that endometriosis and adenomyosis are closely related disorders, and their pathogenesis, involving epigenetic, genetic, molecular and biological factors, is very similar. This significant overlap supports the hypothesis that endometriosis and adenomyosis are two phenotypes of the same disease since they have common pathogenesis factors—proliferation, angiogenesis and local inflammation [40,41,42]. The key difference between adenomyosis and endometriosis lies in the mechanism by which the eutopic endometrium invades the myometrium. In this connection, it seems important to refer to the “archimetra” theory, which is a novel concept in the pathogenesis of adenomyosis. Archimetra is defined as the endometrial–sub-endometrial (also called the junctional zone or stratum subvasculare) unit [43]. A distinct characteristic of the archimetra is the presence of progenitor/stem cells in the basal layer of the endometrium, which possess a high proliferative and invasive potential [44]. This concept elucidating the pathogenesis of adenomyosis posits that patients exhibit myometrium hypercontractility (particularly pronounced during menstruation), which leads to trauma of the basal layer and the junctional zone. As a result, the endometrial stem/progenitor cells contained in the basal layer can penetrate the myometrium. Once inside the myometrium, these misplaced cells begin to proliferate, forming adenomyosis lesions [45,46]. However, all the follow-on pathogenetic patterns of adenomyosis will be the same as the “classical” mechanisms described in the previous sections of our review. To summarize, adenomyosis is one of the phenotypes of endometriosis. It is characterized by the invagination of the eutopic endometrial glands and stroma in the muscular wall of the uterus. Thus, adenomyosis and endometriosis share many common pathogenetic pathways, except those at the initial stage. In adenomyosis, trauma to the endometrial–myometrial junction is a key initial event leading to the invasion of progenitor endometrial cells into the myometrium.

Based on the above, it is important to note that ovarian endometriosis and adenomyosis share significant similarities in the underlying pathophysiology of the ectopic endometrium. However, the impact of these endometriosis phenotypes on the eutopic endometrium is less clear-cut due to proven pathogenetic differences. The differences in the pathogenesis of endometrial dysfunction in ovarian endometriosis and adenomyosis will be discussed below.

### 3.3. Endometriosis-Induced Endometrial Dysfunction—Pathogenetic Mechanisms and Factors

#### 3.3.1. Ovarian Endometriotic Cysts

Recent research provides strong evidence for the pathogenetic interplay between ovarian endometriotic cysts (OECs) and endometrial dysfunction. As shown, OECs and other forms of endometriosis may affect endometrial homeostasis. Moreover, the dysfunctional eutopic endometrium is associated with the pathogenesis of endometriosis (including OECs). The study of Russian researchers which included 172 patients with endometrioid ovarian cysts demonstrated that in the middle stage of the proliferation phase, there was an increase in the levels of expression of estrogen receptor 1 (ER1) in the glands (*p* = 0.0024) and progesterone receptors (PRs) in the stroma (*p* = 0.0000) and glands (*p* = 0.0003) of the endometrium. In addition, the number of pinopodia on the surface of endometrial cells was reduced. Following surgical treatment, the endometrial receptivity and implantation potential progressively improved in these patients, indicating that external genital endometriosis (ovarian type) has a significant pathogenetic influence on endometrial dysfunction [47]. In this review of the core pathogenetic factors and mechanisms of endometriosis, the authors have hypothesized that epigenetic changes are a critical regulatory component. These epigenetic modifications play a central role in the development of heterotopias and may alter the eutopic endometrium. The research by Zhang, R. et al. published in 2025 describes the successful construction of a new in vitro organoid model, using both eutopic and ectopic endometrial tissues from patients with ovarian endometriosis [14]. Genetic analysis revealed a 100% match between endometriosis epithelial organoids and endometrial tissue, indicating a common origin. However, it was not possible to identify the primary source of heterotopias (retrograde menstrual reflux, epithelial metaplasia or the development of endometriosis from Müllerian duct remnants) using this new organoid model [14]. Although the findings do not reveal the primary origin of endometrioid heterotopias, they suggest that genetically induced endometrial dysfunction can be linked to abnormal expression of genes shared between the lesions and the eutopic endometrium.

It should also be noted that eutopic and heterotopic endometrium are considered to have the same genetic identity but display differences in epigenetic changes. The evidence has shown that in patients with endometriosis, epigenetic modifications cause a critical shift in the activity of crucial regulatory proteins GATA2, GATA6, and steroidogenic factor 1 (SF1). Recent research demonstrates that the *GATA2* gene is hypomethylated in the endometrium. In turn, the GATA2 protein increases the expression of genes involved in the decidual transformation and has a function of progesterone mediator. Thus, hypomethylation impairs the decidual transformation and implantation window formation due to progesterone resistance and the decreased expression of progesterone receptors (PRs). In addition to regulating implantation, progesterone has local immunosuppressive effects, preventing the maternal immune system from rejecting the blastocyst, but in endometriosis these effects of progesterone are lost. At the same time, the overexpression of GATA6 and SF1 plays a crucial role in converting cholesterol to metabolically active estradiol, potentiating the implantation failure of the endometrium [16].

Both local and systemic pro-inflammatory patterns are crucial in the development of endometriosis-associated dysfunction. In this context, the study of Nahdi S. et al. (2025) has demonstrated that interleukin-6 (IL-6) is a critical pro-inflammatory factor involved in regulating numerous signaling pathways, including those associated with tumor necrosis factor (TNF) and the IL6/JAK-STAT3 pathway [17]. Thus, IL-6 regulates the expression of its associated genes (*ICAM1, TNFRSF21, CXCL8, DUSP5, HAS2, HBEGF, HIF1A, CASP7, IL6*). The evidence also indicates that genes such as *ICAM1, HAS2*, and *HBEGF* are linked to extracellular matrix remodeling and endometrial dysfunction, while *HIF1A* and *CASP7* are tied to hypoxia and ovarian follicle apoptosis [17].

The recent research by Thong L. Y. et al. (2025) demonstrated that the AUC obtained from the best-performing methylation risk score (MRS) is 0.6748, derived from 746 DNA methylation (DNAm) sites [48]. In addition, the classification performance of the MRS and polygenic risk score (PRS) combined was consistently higher than the PRS alone [48]. The study has shown that there are DNAm signals independent of common genetic variants or epigenetic factors associated with endometriosis pathogenesis and endometriosis-associated endometrial dysfunction. However, future research is needed to explore these relationships further.

Local immune homeostasis, including the macrophage system, is also essential for the physiological transformation of the endometrium. The count of key immunogenic CD68+ macrophages or CD163+ macrophages increases during different stages of menstrual cycle: from 1–2% in the early stage to 7% in the late proliferative stage and 6–15% in the pre-menstrual stage [49,50]. In the endometrium, most macrophages belong to the M2 subtype. They produce anti-inflammatory cytokines, including interleukin-10 (IL-10) and transforming growth factor-beta (TGF-β). M2 macrophages also play a role in regulating extracellular matrix (ECM) remodeling and angiogenesis, thus supporting the decidual-like reaction. In contrast, the frequency of CD68+IL-10−iNOS+ M1 macrophages in the secretory endometrium is significantly lower. This reduction in M1 macrophages is crucial for fostering endometrial receptivity by creating a favorable immune environment essential for successful blastocyst implantation [51].

Recent research by Shi J. et al. (2025) has revealed that women with external genital endometriosis (EGE) and infertility have substantial changes in the counts of various subpopulations of immunogenic cells in the eutopic endometrium [52]. Compared to healthy controls, women from this cohort in the proliferative phase of the menstrual cycle exhibited higher numbers of M1 macrophages, CD8+ T cells, T helper 1 (Th1) cells, T-helper 17 (Th17) cells and γδT cells, while regulatory T cells (Treg) were not determined. In the secretory phase, the patients showed an increase in NK cells, uNK progenitor cells, M1 macrophages, CD8+ T cells, Th1 cells, and Th17 cells, as well as a reduced number of M2 macrophages and a lack of Treg in the endometrium (Table 1) [52].

Thus, in women with infertility related to EGE (including ovarian localization), the altered populations of various immunogenic cells in the endometrium disrupt local immune homeostasis, induce endometrial dysfunction and impaired decidualization, and disturb the formation of the implantation window. Another important player in the processes of endometrium remodeling, decidualization, and implantation is Syndecan-1 (SDC-1). Recent research has shown that SDC-1 acts as a co-receptor, which facilitates the signaling of the essential chemokine CXCL1. Its expression is regulated by IL-1β in decidual tissue during the early stages of gestation [53,54]. These molecular factors play pivotal roles in maintaining immune homeostasis in the endometrium. Particularly, CXCL1 is a potent chemoattractant for immune cells—leucocytes, NK cells, granulocytes and macrophages. The recruitment of these immune cells creates a favorable environment for the implantation of a semi-allogeneic embryo and prevention of early pregnancy losses. CXCL1 is also a known regulator of local angiogenesis which plays an essential role in successful implantation of the embryo into the receptive endometrium [55]. Thus, Syndecan-1 (SDC-1) is one of the essential biological factors involved in regulating immune and angiogenic homeostasis in the endometrium. In recent study, Freitag, N. et al. (2022) described changes in the eutopic endometrium in women with endometriosis-associated infertility [56]. The study revealed that a higher concentration of macrophages coincided with an elevated number of uterine natural killer cells or plasma cells. In addition, patients with endometriosis showed higher endothelial expression of VEGF-A. The study also demonstrated the absence of stromal expression of SDC-1, which is crucial for the physiological process of embryo implantation. Furthermore, the lack of proper SDC-1 function (remodeling of immunogenic and angiogenic profiles of the endometrium) was associated with endometrial dysfunction and implantation failures at early stages [56].

A no less important contribution to endometrial dysfunction in women with ovarian endometriosis is made by the altered expression of cadherins—key regulators of the process of embryo implantation. E-cadherin is an important mediator of adhesion in epithelial cells, including both the endometrium and the trophoblast. Studies demonstrate that E-cadherin is essential for the process of the gestational sac attaching to the uterine wall. It is believed to be an important marker for assessing endometrial receptivity [57]. In contrast, N-cadherin is strongly expressed in both stromal and endothelial cells and plays a central role in mediating cell-to-cell adhesion. This mediator is involved in maintaining microvascular stability and facilitating tissue neoangiogenesis, which are important for the implantation process [58]. Interesting data were received in a laboratory study (2021) demonstrating that E-cadherin was predominantly expressed during the pre-receptive stages and that its expression was significantly reduced during the receptive and post-receptive stages. The authors described a dynamic and specific pattern of cadherin expression for the successful implantation process in the uterus which is characterized by a gradual reduction in E-cadherin expression and an increase in N-cadherin expression. In the same mouse models, failed endometrial receptivity showed upregulation of E-cadherin and downregulation of N-cadherin [59]. The animal model findings align with the data collected in clinical settings. The study of Yun B. S. et al. (2024) showed that in women with ovarian endometrioma on days 19–24 of the menstrual cycle (the implantation window), the relative expression of E-cad protein was increased, being 2.52-fold higher than that of the controls (*p* = 0.013), while the relative expression of N-cad protein was low, being 0.55-fold higher than that of the controls (*p* = 0.030) [60]. Thus, emerging data suggest that ovarian endometriomas contribute to immune dysregulation. In addition, they may be associated with the altered production of epigenetic messengers and crucial genetic markers, dysregulated steroid receptor signaling, endometrial immune dysfunction, and the aberrant expression of the local factors required for successful implantation.

#### 3.3.2. Adenomyosis

It should be noted that external genital endometriosis (including the ovarian type) affects the endometrium and induces reproduction failures in a distant way, while the close anatomical location of adenomyosis to the endometrium leads to faster and more severe localized effects. However, no significant differences are observed in the pathogenetic features of endometrial dysfunction in endometriosis and adenomyosis. Adenomyosis-associated implantation failure is characterized by anatomical and histological deformations in the uterine tissues, compromising embryo implantation. This condition involves some other molecular and biological factors described below.

The recent research of Mitra I. et al. (2025) was focused on the investigation of target gene associations in the eutopic endometrium in patients with adenomyosis and ovarian endometriosis [61]. The study determined 23 significant differentially expressed genes (DEGs) that were found to be common between adenomyosis and endometriosis datasets. The authors also identified *MMP7*, *MMP11*, *IGFBP5*, *SERPINA1*, and *THBS1* as candidate hub genes expressed in both adenomyosis and endometriosis. These genes played a central role, as they formed a tightly connected sub-network of nodes and edges. In addition, *MMP9* and *TIMP1* exhibited a strong association with the hub gene network. A comparison of gene expression levels among adenomyosis and endometriosis has revealed distinct expression patterns: *MMP9* and *MMP7* showed strong discrimination for adenomyosis vs. endometriosis. Furthermore, *MMP7* expression positively correlated with uterine volume, while MMP11 inversely correlated with the myometrial wall thickness ratio in adenomyosis [61]. Not less interesting are the results of recent research conducted in 2024. The researchers demonstrated that in patients with adenomyosis during the mid-secretory phase of the menstrual cycle (the implantation window), upregulated genes included *OLFM1*, *FXYD5*, and *RUNX2*, which are involved in impaired endometrial receptivity, while downregulated genes included *RRM2, SOSTDC1*, and *CHAC2*, which are implicated in recurrent implantation failure. In the gestational phase, in patients from the same cohort, upregulated *CXCL14* and *CYP24A1* and downregulated progesterone receptors (PGRs) were related to pregnancy loss [62]. Based on the research results, we can suggest that the genetic landscape of adenomyosis-associated endometrial dysfunction is highly complex, involving a mix of factors that remain only partially understood. Recent advanced studies have identified numerous genetic loci, as well as gene hubs, associated with endometrial dysfunction. However, further research is needed to generate a broader picture and to answer several critical specific questions.

In the previous sections, the authors reviewed the physiological patterns of immune homeostasis in the endometrium, facilitating its healthy remodeling during the menstrual cycle, and described critical disruptions of the endometrial immune status in women with EGE and implantation failures/infertility. Accumulating evidence suggests that adenomyosis is associated with an imbalance in immune cell populations. Moreover, there are significant differences in the immune cell profiles and specific cell ratios between patients with adenomyosis and those with EGE. As highlighted above [52], in contrast to EGE, the proliferative phase of adenomyosis is characterized by an increase in M2 macrophages and a decrease in the M1 type, while other parameters of both conditions seem to be similar. However, M2 macrophages displayed a higher number, while the number of M1 macrophages was lower in the secretory phase of adenomyosis compared to those in EGE (Table 2).

Finally, adenomyosis is characterized by the altered expression of adhesion molecules such as glycodelin, integrin family members, mucin-1, and osteopontin which significantly disrupts the endometrial environment. These molecular changes hinder the interaction between the embryo and the maternal endometrium. Studies also suggest that aberrant formation of microvilli in the apical endometria may be involved in the negative fertility outcomes associated with adenomyosis, including disturbed reception and further implantation [63].

In summary, patients with adenomyosis exhibit specific genetic aberrations and significant alterations in endometrial immune cell ratios when compared to the endometrium of healthy, disease-free women. In addition, the macrophage profile differs significantly between adenomyosis lesions and the eutopic endometrium in patients with EGE. Studies have also shown that aberrant expression of intercellular adhesion molecules contributes to endometrial dysfunction in women with adenomyosis. These identified changes may underlie endometrial dysfunction and play a significant role in implantation failure in women with adenomyosis.

**Figure 2:**
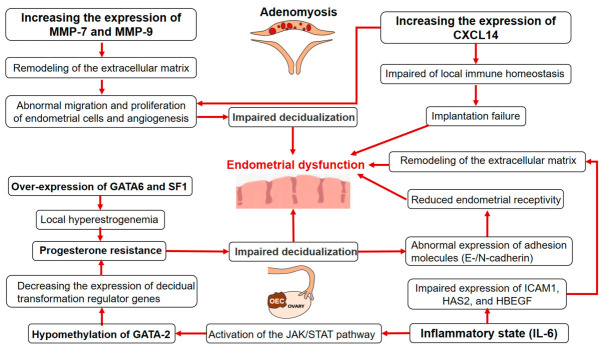
Key pathogenetic pathways of endometrial dysfunction associated with ovarian endometriosis and adenomyosis.

Figure 2 shows the key pathogenetic mechanisms of development of endometrial dysfunction induced by ovarian endometriosis and adenomyosis.

## 4. Conclusions

**Figure 3:**
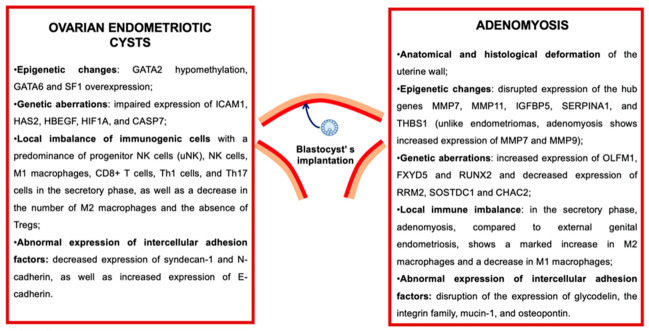
Pathogenetic differences in endometrial dysfunction associated with ovarian endometriosis or adenomyosis.

Endometriosis is still a “mysterious” disease, representing a substantial medical and social burden. It is suggested that endometriosis, across its various phenotypes (adenomyosis, superficial peritoneal endometriosis, ovarian endometriosis, or deep infiltrative endometriosis), adversely affects the endometrium in a multifaceted manner, inducing endometrial dysfunction, which ultimately contributes to reproduction failures, particularly at the early stages of implantation (Figure 3). Moreover, the pathogenesis of endometrial dysfunction in various forms of endometriosis differs fundamentally and has not been compared to date. Therefore, in this article, we aimed to summarize the available data on the basic similarities and differences in the pathophysiology of ectopic endometrial lesions in these disease phenotypes and highlight the “parallel” differences in the mechanisms underlying eutopic endometrial dysfunction in these conditions. Though the authors have highlighted several complex issues and various standpoints relating to endometrial dysfunction associated with adenomyosis and ovarian endometriosis, there are still many unresolved questions and conflicting versions surrounding this problem. Therefore, further investigation is needed to create a comprehensive pathogenetic pattern of endometrial dysfunction.

Furthermore, determining whether endometrial dysfunction is a primary trigger (Sampson’s theory) or a secondary consequence of different endometriosis phenotypes (particularly ovarian endometriomas or adenomyosis) is a central, yet unresolved, issue. Although the molecular patterns of the eutopic endometrium have been described in the scientific literature and our review article, their ultimate role remains an open question. Apparently, a dissociation of local molecular biological processes (epigenetically or genetically induced) in the eutopic endometrium may lead to abnormal implantation of endometrial cells in different anatomical compartments during retrograde menstruation. Therefore, this issue is now a critical research priority. Further studies are needed to define the pathogenetic links between different forms of endometriosis and endometrial dysfunction and to establish the chronology and casual relationship.

## Figures and Tables

**Table 1:** Comparison of immune cell counts in the intact endometrium and the eutopic endometrium in women with EGE [52].

Cells	Intact Endometrium		Eutopic Endometrium in EGE	
	Proliferative Phase	Secretory Phase	Proliferative Phase	Secretory Phase
M1 macrophages	+	+	++	++++
M2 macrophages	+	++	+	+
uNK	+	+	+	++
NK	++	++++	++	+++++ *
CD8+ T cells	+	+	+++	+++
Th1	+	+	++	++
Th2	+	+	+	+
Th17	+	+	++	++
Treg	+	++	-	-
γδT	+	++	++	++

* NK cells without killer Ig-like receptors (KIRs). The number of “+” reflects the number of immune cells: “-”—absent, “+”—present in small quantities, “++”—present in moderate quantities, “+++”—present in average quantities, “++++”—present in large quantities, “+++++”—high degree of saturation in the tissue.

**Table 2:** Comparison of immune cell numbers in adenomyosis and EGE [52].

Cells	The Endometrium in Adenomyosis		The Eutopic Endometrium in EGE	
	Proliferative Phase	Secretory Phase	Proliferative Phase	Secretory Phase
M1 macrophages	+	+	++	++++
M2 macrophages	++	++++	+	+
uNK	+	++	+	++
NK	++	+++++ *	++	+++++ *
CD8+ T cells	+++	+++	+++	+++
Th1	++	++	++	++
Th2	+	+	+	+
Th17	++	++	++	++
Treg	-	-	-	-
γδT	++	++	++	++

* NK cells without killer Ig-like receptors (KIRs). The number of “+” reflects the number of immune cells: “-”—absent, “+”—present in small quantities, “++”—present in moderate quantities, “+++”—present in average quantities, “++++”—present in large quantities, “+++++”—high degree of saturation in the tissue.

## Data Availability

No new data were created or analyzed in this study.

## Abbreviations

The following abbreviations are used in this manuscript:

SPE	Superficial peritoneal endometriosis
DIE	Deep infiltrating endometriosis
EGE	External genital endometriosis
VEGF	Vascular endothelial growth factor
OECs	Ovarian endometriotic cysts
PGR	Downregulated progesterone receptor
